# ROS Homeostasis Involved in Dose-Dependent Responses of *Arabidopsis* Seedlings to Copper Toxicity

**DOI:** 10.3390/genes14010011

**Published:** 2022-12-21

**Authors:** Jiehua Wang, Muhammad Moeen-ud-din, Rong Yin, Shaohui Yang

**Affiliations:** School of Environmental Science and Engineering, Tianjin University, Tianjin 300072, China

**Keywords:** *Arabidopsis thaliana*, copper (Cu), reactive oxygen species (ROS), DNA damage response (DDR), dose-dependency, gene expression

## Abstract

As an essential element in plant nutrition, copper (Cu) can promote or inhibit plant growth depending on its concentration. However, the dose-dependent effects of copper, particularly on DNA damage associated with reactive oxygen species (ROS) homeostasis, are much less understood. In this work, we analyzed the dual effect of Cu (5, 20, and 60 μM) on the reproductive performance of Arabidopsis plants. Whereas Cu5 promoted inflorescence initiation and increased kilo seed weight, two higher concentrations, Cu20 and Cu60, delayed inflorescence initiation and negatively affected silique size. Excess Cu also induced changes in cellular redox homeostasis, which was examined by in situ visualization and measurements of ROS, including superoxide (O_2_^•−^), hydrogen peroxide (H_2_O_2_), malonyldialdehyde (MDA), and plasma membrane damage. The most dramatic increases in the production of O_2_^•−^ and H_2_O_2_ along with increased activity of superoxide dismutase (SOD) and glutathione peroxidase (GPX) and decreased activity of catalase (CAT) and ascorbate peroxidase (APX) were observed in roots with Cu60. Oxidative stress also modulated the expression levels of a number of genes involved in the DNA damage response (DDR), particularly those related to DNA repair. The Cu-induced chlorosis of Arabidopsis seedlings could be alleviated by exogenous addition of glutathione (GSH) and ascorbate (Asc), as the chlorophyll content was significantly increased. Overall, internal homeostasis ROS and the associated DDR pathway and the corresponding scavenging mechanisms play a central role in the response of Arabidopsis to oxidative stress induced by inhibitory Cu concentrations. Our results have shown, for the first time, that the biphasic responses of Arabidopsis seedlings to increasing Cu concentrations involve different DNA damage responses and oxidative reactions. They provide the basis for elucidating the network of Cu-induced DDR-related genes and the regulatory mechanism of the complex ROS production and scavenging system.

## 1. Introduction

As an essential micronutrient for plants, copper (Cu) is an important cofactor for many metalloproteins and plays a key role in important biological processes such as respiration, photosynthesis, antioxidant activity, cell wall metabolism and lignification, and ethylene signaling [1,2]. However, because it is a transition metal with high redox activity, Cu excess can be toxic to plants and, in particular, can catalyze the formation of harmful free radicals that disrupt numerous biochemical and physiological processes [2,3]. Symptoms of Cu excess in plants include reduced plant biomass, inhibited root growth, and necrosis–chlorosis [2]. Previously, we showed that the phenotypic responses of Arabidopsis seedling roots were dose-dependent, which correlated well with the change in the concentration of endogenous auxin [4]. In the current study, we continue to investigate plant adaptation strategies and mechanisms of Cu in the context of cellular redox homeostasis.

As a redox-active metal, Cu catalyzes the formation of reactive oxygen species (ROS) through the Fenton and Haber–Weiss reactions [1]. Therefore, excess Cu often leads to the accumulation of harmful ROS, including superoxide (O_2_^•−^), hydrogen peroxide (H_2_O_2_), and hydroxyl radical (^•^HO), which disrupt redox homeostasis and cause a variety of oxidative damage to cells at the lipid, protein, and nucleic acid levels [5,6,7]. For example, as a relatively long-lived molecule, H_2_O_2_ can diffuse from the site of production and penetrate membrane structures to induce intracellular damage [8]. The increased Cu tolerance in autotetraploid Arabidopsis compared to diploid wild-type Arabidopsis has been linked to its enhanced antioxidant activity [9], and the Arabidopsis *miox4* mutant also exhibits higher resistance to deleterious Cu with reduced ROS production [6]. It has been widely reported in the literature that in order to survive and adapt to oxidative damage caused by excessive metal ions, plants have evolved an intricate defense network consisting of ROS-removing enzymes such as superoxide dismutase (SOD), ascorbate peroxidase (APX), catalase (CAT), glutathione reductase (GR), and peroxidase (POD) [10,11,12], as well as low molecular mass antioxidants such as ascorbate (Asc) and glutathione (GSH) [11]. The sequential and additive action of these antioxidant enzymes and compounds constitutes the antioxidant force to maintain cellular redox homeostasis. As previously shown, Cu stress induces the overproduction of the enzymes SOD, APX, and CAT in *Ceratophyllum demersum* [13] and the activities of CAT, POD, and APX in the duckweed *Lemna minor* [14].

The accumulation of ROS also causes oxidative damage to DNA molecules and subsequently leads to impaired genomic integrity and stability [15]. To defend against DNA damage, plants initiate a DNA damage response (DDR) network, which consists of a series of DNA damage detection and signal transduction pathways [16], to arrest the cell cycle and even trigger cell death [17,18]. The DDR network is a highly conserved system in various eukaryotic species, and in plants, a number of proteins such as WEE1 (Wee1-like protein kinase) [19], MRE11 (meiotic recombination 11) [20,21], PARP1 (poly(ADP-ribose) polymerase 1) [22], and BRCA1 (breast cancer susceptibility1) [23] have been shown to play important roles in DDR responses to maintain genome stability.

In the literature, a number of authors have described the responses of plants when exposed to relatively high and acute concentrations of copper [24,25], but there is far less research on the dose-dependent effects of copper, particularly on DNA damage related to ROS homeostasis. In this work, we aimed to (1) investigate the involvement of cellular redox balance in determining the tolerance of Arabidopsis seedlings to different doses of Cu by analyzing the changes in antioxidant enzyme activities and transcriptional changes in the genes encoding them; (2) evaluate the DNA damage response in Cu-stressed Arabidopsis seedlings by analyzing the accumulation of ROS and the expression levels of various DDR marker genes; (3) analyze the ameliorative effect of antioxidant Asc and GSH on Cu toxicity in Arabidopsis seedlings. Our data presented here may help elucidate the dose-dependent effects of Cu on plants in relation to cellular redox status and shed light on the development of Cu-tolerant germplasm for sustainable agricultural systems. In addition, the study may provide the basis for elucidating the network of Cu-induced DDR-related genes and the regulatory mechanism of the complex ROS production and scavenging system.

## 2. Materials and Methods

### 2.1. Plant Material, Growth Conditions, and Cu Treatment

Seeds of wild-type Arabidopsis thaliana (ecotype Col-0) were surface-sterilized with 12.5% sodium hypochlorite for 7 min and with 70% ethanol for 2 min and then washed 5 times with sterile water. After 3 days of vernalization at 4 °C in the dark, seeds were grown on 1/2 Murashige and Skoog (MS) medium (with 0.05 µM Cu) at pH 5.7 consisting of 1% (*w/v*) sucrose, 0.8% (*w/v*) phytoagar, and 0.05% 2-(4-morpholino) ethanesulfonic acid (MES) supplemented with CuSO_4_ at an additional concentration of 0, 5, 20, or 60 µM (control, Cu5, Cu20, or Cu60).

Seedlings were grown vertically in a growth chamber at a day/night temperature of 22 °C/18 °C, 16 h light/8 h dark, and a light intensity of 120 µmol m^−2^ s^−1^. After 6 days of growth, more than 30 plants per treatment were repotted into a 1:1 (*v/v*) perlite-vermiculite mixture soaked in 1/2 MS liquid medium with appropriate CuSO_4_ concentration. Pots were kept in plastic bags to prevent the loss of Cu. Plants were watered once a week with an appropriate amount of distilled water to keep the soil moist. The pot culture experiment was conducted in a greenhouse at a temperature of 21 ± 1 °C, a photoperiod of 16 h of light with a light intensity of 140 µmol m^−2^ s^−1^, and a relative humidity of 50%. In the pot experiment, the indices of reproductive growth of plants were evaluated, including the height of plant stems, time of bolting, length of siliques, and weight of one thousand seeds of Arabidopsis treated with different concentrations of CuSO_4_. For the experiment to test the function of Asc and GSH, seeds were sown on 1/2 MS plates containing 60 µM CuSO_4_ with or without 0.3 mM Asc or 1 mM GSH, and total chlorophyll content was determined after 6 days.

### 2.2. Determination of Physiological Indices

Arabidopsis seeds were germinated on 1/2 MS medium containing various concentrations of CuSO_4_ and grown for 6 days before shoots or roots were harvested separately for subsequent analysis. Fresh leaves (100 mg) were pulverized with liquid nitrogen, and chlorophyll was extracted with 95% (*v/v*) ethanol. Extraction was carried out at 4 °C for 2 h with the exclusion of light. The extract was centrifuged at 8000× *g* for 5 min at 4 °C. The absorbance of the supernatant at a wavelength of 652 nm was determined using a spectrophotometer (RAY LEIGH UV-1801,Beijing, China). The total chlorophyll content was calculated according to our previous study [26]. Membrane integrity was assessed by measuring the electrolyte leakage of the leaf using a previous method [27]. Quantitative detection of H_2_O_2_, O_2_^•−^, MDA, GSH, and Asc was performed using assay kits (Zike, Shenzhen, China) according to the manufacturer’s protocol. The enzyme activities of SOD, CAT, APX, and glutathione peroxidase (GPX) were determined using assay kits (Shenzhen Zike Biotechnology Company, Shenzhen, China) according to the manufacturer’s instructions.

### 2.3. Histochemical Localization Analysis

In situ visualization of O_2_^•−^ was performed by using nitroblue tetrazolium (NBT) staining as previously described by Bournonville and Diaz-Ricci [28]. Briefly, 6-day-old seedlings were randomly selected in triplicate from different concentrations of CuSO_4_ treatments. Root and shoot samples were vacuum-filtered for 2 min in 50 mM phosphate buffer (pH 7.6) containing 0.1% NBT and 10 mM sodium azide, and then incubated for 2 h in the dark. Finally, they were washed once with phosphate buffer and immersed in 96% ethanol to completely remove chlorophyll.

Intracellular H_2_O_2_ was visually detected by 3, 3-diaminobenzidine (DAB) staining, a method described by Guan et al. [29]. Root and shoot samples from 6-day-old seedlings were incubated in 0.1% DAB staining solution in distilled water for 8 h in the dark. Samples were washed at least three times with distilled water and then bleached with (1:1:3) acetic acid: glycerol: ethanol to remove chlorophyll. The loss of membrane integrity or cell death was stained with trypan blue as described by Duan et al. [30]. Six-day-old seedlings were incubated in a 0.4% trypan blue solution for 20 min and then washed three times with distilled water. In all cases, the seedlings were immediately mounted on microscopic slides and histochemical staining was observed using Nikon microscope 50i equipped with a Nikon DS -Fi1C camera. The images were analyzed using Image J software.

### 2.4. RNA Isolation and qRT-PCR Analysis

The expression of genes encoding antioxidant enzymes was investigated by qRT-PCR according to our previous study [26]. The roots and shoots of six-day-old Arabidopsis seedlings were harvested separately and ground in liquid nitrogen to extract total RNA. Total RNA was extracted using an RNeasy Plant Mini Kit (Qiagen) according to the manufacturer’s instructions. The cDNA was prepared using SuperScript III reverse transcriptase (Invitrogen, Thermo Fisher Scientific, USA). qRT-PCR Reactions were performed using SYBR Premix Ex Taq II (Takara, Dalian, China) according to our previous study [26]. The relative expression of target genes was calculated using the ^ΔΔ^Ct method [31]. The constitutively expressed actin gene (*AtActin*) was used as an internal control. The sequences of all primers used for qRT-PCR in this study are listed in Table S1.

### 2.5. Statistical Analysis

All experiments in this study were repeated three times. Values shown are means ± SD of three individual experiments. Statistical significance was determined by a one-way ANOVA/LSD post hoc test (*p* < 0.05). The analysis of all data and the drawing of graphs were performed using SPSS, Microsoft Excel, and Visio software.

## 3. Results and Discussions

### 3.1. Reproductive Performance of Arabidopsis upon Cu Treatment

Our previous work showed that wild-type Arabidopsis seedlings grew optimally in 1/2 MS medium supplemented with a CuSO_4_ concentration of 5 μM, and increasing concentrations had dose-dependent negative effects [32]. Therefore, 20 μM Cu (Cu20) was established as the threshold for Cu toxicity, and 60 μM Cu (Cu60) as the dose causing severe adverse effects [5]. Considering that Cu deficiency primarily affects the reproductive performance of higher plants [33], we first investigated how the above three Cu doses affected the reproductive parameters. The results showed that the three Cu doses had different effects on reproductive performance, except for inflorescence stalk height. The bolting time was 3 days earlier and 6 days later for Cu5 and Cu60, respectively, than the control plants (Table 1). The average length of siliques was 16% lower in Cu60-treated plants than in untreated control plants (Table 1). In terms of kilo seed weight, Cu5 resulted in an increase of 15%, whereas Cu20 and Cu60 resulted in a comparable seed weight to the control plants (Table 1). Together with the previous reports focusing on the vegetative stages of Arabidopsis seedlings, the results confirmed that the effect of copper on Arabidopsis thaliana is dose-dependent.

### 3.2. Dose-Dependent Accumulation of ROS Induced by Cu in Arabidopsis Seedlings

**Figure 1:**
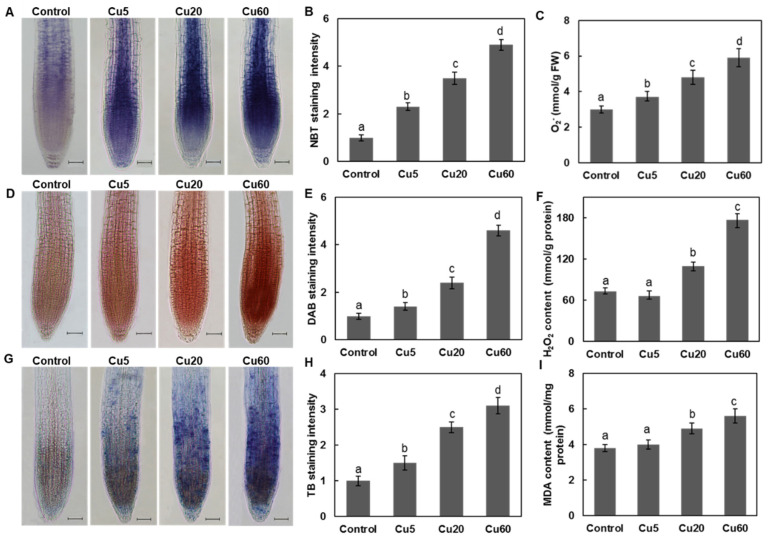
Accumulation of reactive oxygen species (ROS) and oxidative damage in roots of Arabidopsis seedling after treated with different concentrations of CuSO_4_ for 6 d. (**A**) Nitroblue tetrazolium (NBT) staining for superoxide anion. (**B**) Relative NBT staining intensity shown in (**A**). (**C**) O_2_^−^ contents. (**D**) 3, 3-diaminobenzidine (DAB) staining for H_2_O_2_. (**E**) Relative DAB staining intensity shown in (**D**). (**F**) H_2_O_2_ contents. (**G**) Trypan blue (TB) staining. (**H**) Relative TB staining intensity shown in (**G**). (**I**) Malonyldialdehyde (MDA) levels. The relative color intensity of control was set as 1. At least ten images from three different experiments were measured. Data are means ± SD of three replicates with 30 seedlings. All the treatments were performed three times. Different letters indicate the significant difference (*p* < 0.05, by ANOVA-LSD post hoc test). Scale bar = 50 µm.

**Figure 2:**
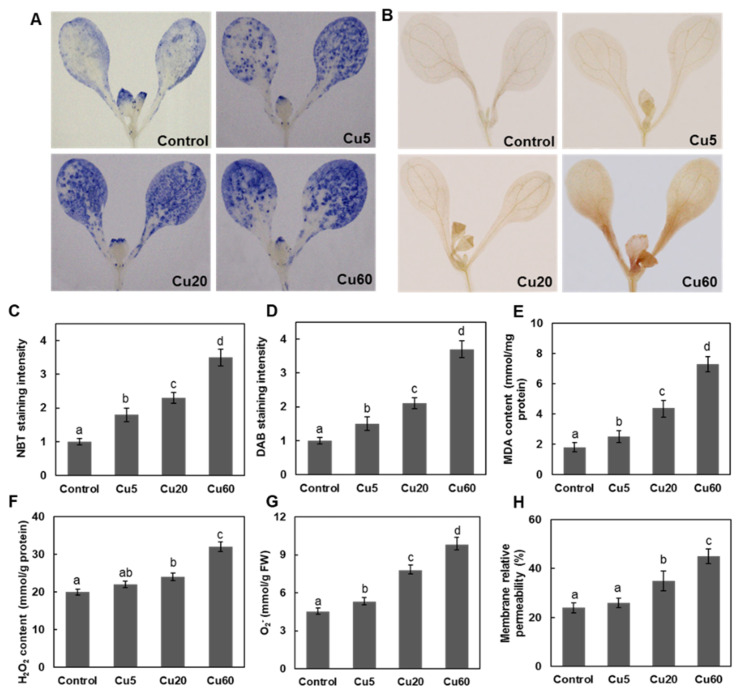
Accumulation of ROS and oxidative damage in shoots of Arabidopsis seedling after treated with different concentrations of CuSO_4_ for 6 d. (**A**) NBT staining for superoxide anion. (**B**) DAB staining for H_2_O_2_. (**C**) Relative NBT staining intensity shown in (**A**). (**D**) Relative DAB staining intensity shown in (**B**). (**E**) MDA levels. (**F**) H_2_O_2_ contents. (**G**) O_2_^−^ contents. (**H**) Plasma membrane permeability of leaves. The relative color intensity of control was set as 1. Data are means ± SD of three replicates with 30 seedlings. All the treatments were performed three times. Different letters indicate the significant difference (*p* < 0.05, ANOVA-LSD post hoc test).

Abiotic stress always disturbs cellular redox homeostasis and increases the production of ROS, so we investigated the levels of O_2_^•−^ and H_2_O_2_ in Arabidopsis tissues after Cu treatments by histochemical and biochemical analyses. In both roots and shoots, three Cu concentrations triggered O_2_^•−^ and H_2_O_2_ production in a dose-dependent manner, as shown by the NBT and DAB staining assays, respectively (Figure 1 and Figure 2). As for H_2_O_2_ production, a dramatic increase in DAB staining intensity in roots was observed only for Cu20 and Cu60 compared with untreated control plants (Figure 1E and Figure 2D). The modulation of ROS content in roots (Figure 1C,F) and shoots (Figure 2F,G) was verified by biochemical measurements, which was consistent with previous reports. When 3-week-old Arabidopsis seedlings were exposed to 5 μM Cu for 24 h, H_2_O_2_ content in leaves and roots increased by 80% and 86%, respectively [34]. When the growth medium contained 25–50 μM CuSO_4_, Arabidopsis leaves exhibited 440–480% of the H_2_O_2_ concentration of control plants [35]. In leaves of *A. thaliana* treated with 100 μM Cu, H_2_O_2_ content increased 3.6-fold after 48 h and 9.5-fold after 144 h [7]. In conclusion, excessive Cu induces the accumulation of ROS, including O_2_^•−^ and H_2_O_2_, thus disrupting cellular redox homeostasis.

As an intermediate of ROS, H_2_O_2_ removes electrons from the lipids of cell membranes, resulting in severe lipid peroxidation [11]. Therefore, MDA content, one of the end products of lipid peroxidation, was analyzed to evaluate the extent of oxidative damage caused by Cu. In this work, MDA content was significantly increased in both roots (Figure 1I) and shoots (Figure 2E). In roots, oxidative damage to the plasma membrane detected by trypan blue staining also showed a dose dependency and there was a clear indication of cell damage after treatment with Cu20 and Cu60 (Figure 1G,H). In Arabidopsis, the uptake of Cu mainly relies on the high-affinity transporter (COPT1) localized in the plasma membrane of root tip cells [32], where the massive entry of Cu could generate ROS [36]. When treated with 50 µM CuSO_4_ for 2 h, this increased cytosolic Cu pool produces a burst of reactive hydroxyl radicals (^•^OH) in the cytosol of Arabidopsis root tip cells [37]. In shoots, Cu5 did not appear to alter membrane permeability in Arabidopsis seedling leaves (Figure 2H), but Cu20 and Cu60 increased membrane permeability by 45% and 88%, respectively, compared with the control (Figure 2H), suggesting that excess Cu damages this essential physiological function of plant cells.

### 3.3. Cu Induces Oxidative DNA Damage Response in Arabidopsis Seedlings

**Figure 3:**
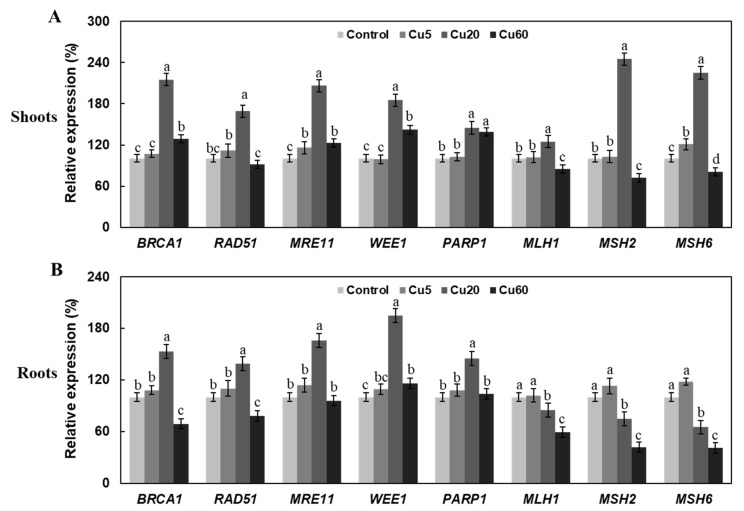
Expression levels of eight DDR-related genes in shoots (**A**) and roots (**B**) of 6-day-old Arabidopsis seedlings exposed to different concentrations of CuSO_4_. Expression levels of Arabidopsis seedlings grown on ½ MS medium served as controls and were set to 100% by qRT-PCR analysis. Data are means ± SD of three replicates. Different letters indicate significant difference between treatments (*p* < 0.05, ANOVA-LSD post hoc test).

Another serious consequence of the accumulation of ROS is oxidative DNA damage, which plants counteract by a series of DDR-related proteins such as MRE11, WEE1, RAD51, PARP1, and BRCA1 to maintain genome stability [16,17]. Since the products of these genes play an important role in the repair of double-stranded DNA breaks (DSBs) or DNA end joining [15], their mutation caused hypersensitivity to irradiation or genotoxic agents [38,39]. To date, little is known about the effects of Cu stress on DDR induction. Therefore, we examined DDR Arabidopsis seedlings after Cu stress by analyzing the transcript levels of eight DDR marker genes, including *AtMRE11*, *AtRAD51*, *AtBRCA1*, AtWEE1, *AtPARP1*, *AtMSH2*, *AtMSH6*, and *AtMLH1*, by qRT-PCR analysis. As shown in Figure 3, the induction of DDR genes by Cu20 was most pronounced compared with Cu5 and Cu60. In both shoots and roots, the transcription of all genes was significantly up-regulated by Cu20, except for *AtMSH2*, *AtMSH6*, and *AtMLH1* in roots (Figure 3A,B). In comparison, Cu5 induced the expression of fewer genes and to a lesser extent, and Cu60 even decreased the expression of several genes in both tissues (Figure 3A,B), suggesting a dysfunctional plant DNA repair system at this high concentration. Similar to our results on Cu stress, the expression of *RAD51*, *BRCA1*, *MRE11*, *WEE1*, *MSH1*, *MSH2*, and *MSH6* was also significantly decreased in Arabidopsis roots under 2.5 and 4.0 mg/L Cd stress [39].

### 3.4. Cu Regulates Antioxidative Enzyme Activity in Arabidopsis Seedlings

**Figure 4:**
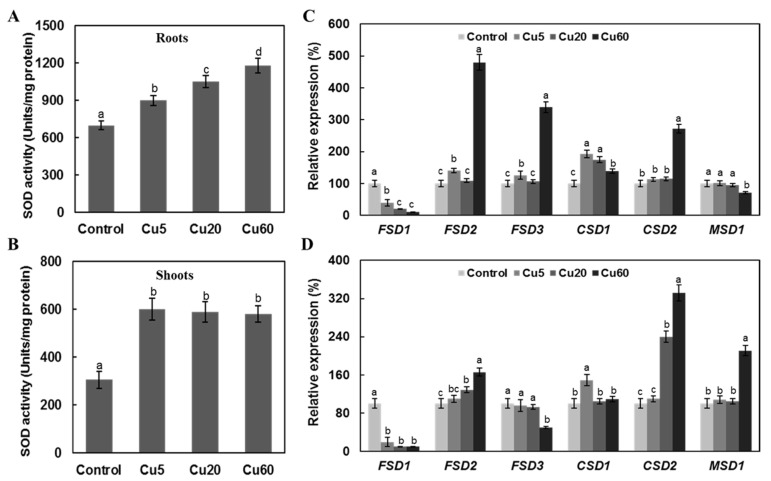
Effect of three different concentrations of CuSO_4_ on total superoxide dismutase (SOD) activity and expression levels of genes encoding multiple SOD isoforms in roots and shoots of 6-day-old Arabidopsis. SOD activities in roots (**A**) and shoots (**B**). Expression levels of genes encoding multiple SOD isoforms (FSDs, CSDs, and MSDs) in roots (**C**) and shoots (**D**). Data are means ± SD of three replicates with 30 seedlings. Different letters indicate a significant difference (*p* < 0.05, ANOVA-LSD post hoc test).

The metabolism of ROS is controlled by a group of enzymes, of which SOD and catalase (CAT) are the most important enzymes to lower the level of ROS and maintain redox homeostasis [12,40]. In Arabidopsis leaves exposed to Cu for 7 days, a gradual increase from 120% of control at 5 μM Cu to 400% of control at 300 μM Cu was reported [35]. However, in two other studies, the total activities of SOD in roots and leaves were not affected when the roots of 3-week-old Arabidopsis seedlings were exposed to 2–5 μM Cu [34] and 10 μM Cu [41] for 24 h via hydroponic nutrient solutions. In barley seedlings, treatment with 15 μM Cu resulted in a slight, but significant, increase in the total activity of SOD, whereas much higher Cu concentrations of 150 and 1500 μM caused visible toxicity symptoms, as evidenced by a decrease in the activity of SOD [42]. In this work, both roots and shoots showed higher SOD activities, with a significant correlation observed between Cu concentration and increased activity of SOD in roots (Figure 4A), whereas the activities of SOD in shoots were similarly upregulated by three doses of Cu (Figure 4B). Such differential responses in tissues with respect to SOD enzyme activities were also observed in 2-week-old Arabidopsis seedlings exposed to γ radiation for 7 days [43].

Copper/zinc SODs (Cu/ZnSODs, CSDs), manganese SODs (MnSODs, MSDs), and iron SODs (FeSODs, FSDs) are three classes of SODs in plants that play an important role in adaptation to abiotic stresses [40]. We then investigated whether or not the change in the overall activity of SOD was associated with changes in the expression levels of genes encoding the different SOD isoforms. First, *FSD1* was found to be the only gene that was strongly down-regulated in both shoots and roots upon Cu exposure. *MSD1* and *FSD3* were moderately down-regulated by Cu in roots (Figure 4C) and shoots (Figure 4D), respectively. All other SOD-coding genes tested were unchanged or up-regulated in their transcription (Figure 4C and Figure 4D). In roots, *FSD2*, *FSD3*, and *CSD2* were among the most Cu60-induced (>2-fold) genes (Figure 4C). In shoots, the expression levels of *CSD2* at Cu20 and Cu60 and of *MSD1* at Cu60 were dramatically higher than in control group plants (Figure 4D). Thus, the Cu-induced increased activities of SOD in Arabidopsis shoots and roots were associated with the increased expression of several SOD genes. In agreement with our results, an induction of *CSD2* and an increase in SOD activity were detected in Arabidopsis leaves in response to 30 µM Cu [44]. In hydroponically grown Arabidopsis leaves, the activities of CSD1 and CSD2 were decreased under Cu-deficient conditions and increased when Cu was added to the medium [45]. In contrast, FSD was up-regulated under copper-deficient conditions, but disappeared when Cu was added [44]. Our results confirmed the contrasting responses of Cu/ZnSOD and FeSOD to different Cu availabilities, suggesting that the superoxide scavenging functions of cytosolic CSD1 in the presence of excess Cu may substitute for the activity of FSD1 localized in plastids.

**Figure 5:**
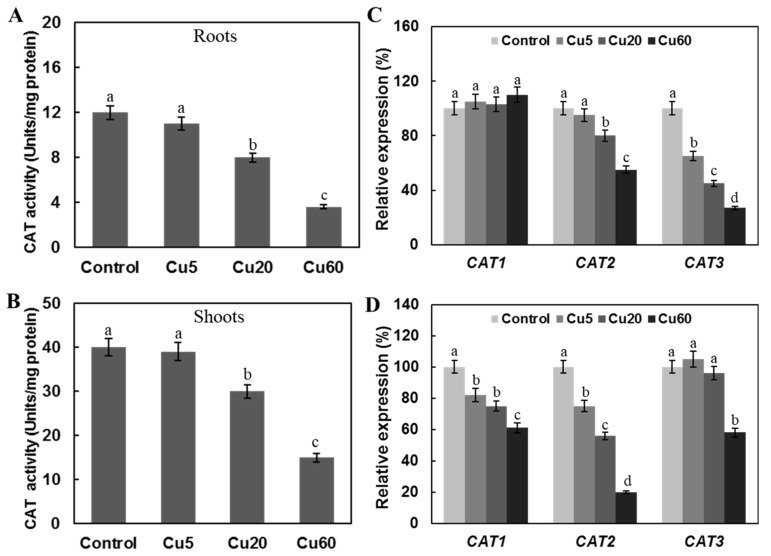
Effect of three different concentrations of CuSO_4_ on catalase (CAT) activity and expression levels of genes encoding CAT in roots and shoots of 6-day-old Arabidopsis. CAT activities in roots (**A**) and shoots (**B**). Expression levels of genes encoding multiple CAT isoforms in roots (**C**) and shoots (**D**). Data are means ± SD of three replicates with 30 seedlings. Different letters indicate the significant difference (*p* < 0.05, ANOVA-LSD post hoc test).

CAT converts H_2_O_2_ to water and molecular oxygen and is considered one of the most important ROS scavengers in peroxisomes [10]. In the leaves of Arabidopsis exposed to Cu for 7 days, it was shown that the activity of CAT gradually decreased with increasing Cu concentration and at 75 μM, Cu was only about 40% of the control values [35]. In another work, the activity of CAT in 11-day-old Arabidopsis seedlings treated with 35 μM CuSO_4_ for three days was 71% and 83% in roots and shoots, respectively [32]. In our study, Cu5 showed no effect on the activity of CAT, but at Cu20 and Cu60, the activities of CAT were significantly decreased in both roots and shoots compared with control plants (Figure 5A,B). qRT-PCR results showed that the decrease in the activity of CAT in shoots and roots was supported by the decreased expression of *CAT1*-*3* genes (Figure 5C,D).

**Figure 6:**
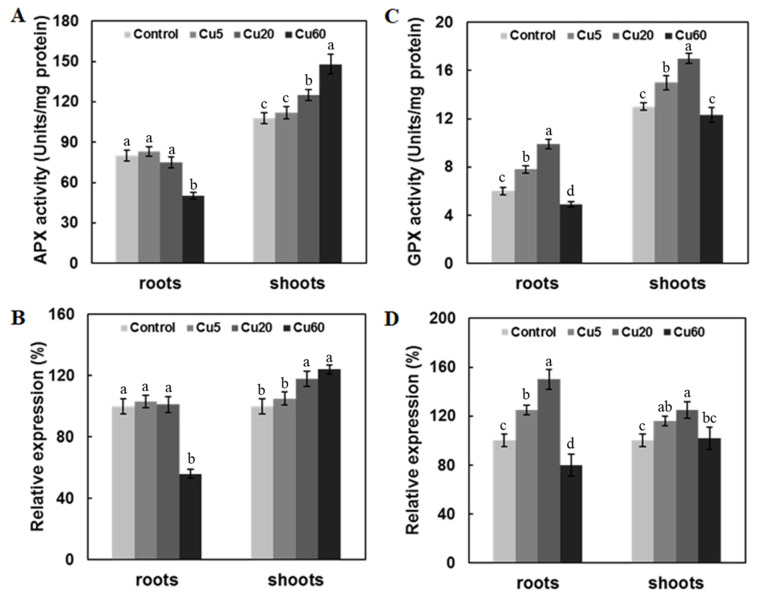
Effect of three different concentrations of CuSO_4_ on ascorbate peroxidase (APX) and glutathione peroxidase (GPX) activity and expression levels of their encoding genes in roots and shoots of 6-day-old Arabidopsis. APX activities (**A**). Expression levels of *APX1* gene (**B**). GPX activities (**C**). Expression levels of *GPX1* gene (**D**). Data are means ± SD of three replicates with 30 seedlings. Different letters indicate the significant difference (*p* < 0.05, ANOVA-LSD post hoc test).

As the first step of the Asc-GSH cycle, ascorbate peroxidase (APX) plays an important role in reducing H_2_O_2_ accumulation by using two molecules of ascorbate to reduce H_2_O_2_ to water [46]. In the current work, the activity of APX in Arabidopsis shoots showed dose-dependent increases at three Cu concentrations and a consistent trend in the expression of the APX gene. However, in roots treated with Cu60, the activity of APX decreased significantly (Figure 6A), along with a drastically decreased expression of *APX1*, which was absent in Cu5 or Cu20 (Figure 6B), suggesting that the increased H_2_O_2_ at high Cu concentrations in roots may be due to the impaired activity of APX, as previously suggested in another work [35].

Similar to APX, GPX plays an essential role in ROS homeostasis and stress signaling in plants and, thus, can be used as a biomarker of oxidative damage [47]. In our study, Cu5 and Cu20 significantly up-regulated GPX activity in both shoots and roots. In contrast, Cu60 down-regulated GPX activity only in roots (Figure 6C). The qRT-PCR results showed that these changes were associated with the modulated expression level of the *GPX1* gene (Figure 6D). Similar to our results, 200 mg/L Cu exposure also reduced the GPX activity of *Belamcanda chinensis* calli from 7 d to 49 d [48]. Thus, the significantly reduced activities of APX and GPX could be attributed to H_2_O_2_ accumulation upon excessive Cu exposure.

### 3.5. Cu-Induced Changes of AsA and GSH Contents in Arabidopsis Seedlings

**Figure 7:**
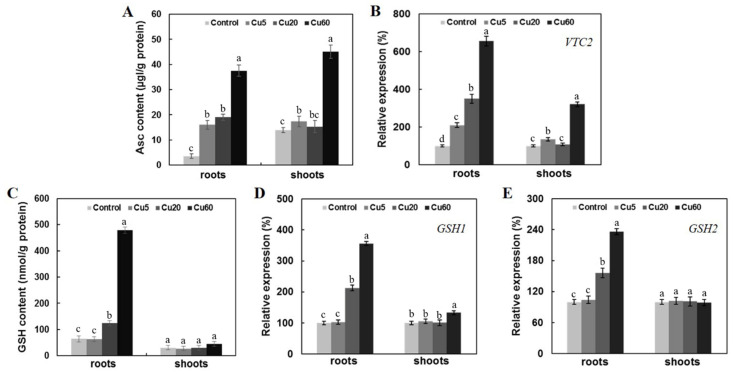
Effect of three different concentrations of CuSO_4_ on ascorbate (Asc) and glutathione (GSH) contents and the expression levels of their encoding genes in roots and shoots of 6-day-old Arabidopsis. Asc contents (**A**). Expression levels of *VTC2* gene (**B**). GSH contents (**C**). Expression levels of *GSH1 and GSH2* gene (**D**,**E**). Data are means ± SD of three replicates with 30 seedlings. Different letters indicate the significant difference (*p* < 0.05, by ANOVA-LSD post hoc test).

Ascorbate (Asc) and glutathione (GSH) are both multifunctional, non-enzymatic metabolites that play a prominent role in redox balance. A high ratio of reduced to oxidized Asc and GSH is essential for ROS scavenging in cells [48]. For Asc synthesis in Arabidopsis, the L-galactose pathway with VTC2 as the rate-limiting enzyme is the major metabolic pathway and the Asc-deficient mutants *vtc2-1* and *vtc2-3* have slightly elevated ROS levels and are Cu-sensitive [6]. When Asc content was measured in shoots and roots of Arabidopsis seedlings under different Cu treatments, Cu60 significantly increased Asc content in both shoots and roots (Figure 7A), which was accompanied by increased *VTC2* gene expression (Figure 7B).

GSH is the predominant non-protein thiol and holds Asc in the reduced form [11,49]. GSH is synthesized by two sequential ATP-dependent reactions catalyzed by GSH1 and GSH2 [48]. GSH level [7] and the abundance of *GSH1* transcripts have been shown to be increased after Cu treatment in Arabidopsis [50]. In this work, Cu60 dramatically increased GSH content in the roots, but not in the shoots, of Arabidopsis seedlings (Figure 7C), and the transcript levels of *GSH1* and *GSH2* genes were up-regulated mainly in roots exposed to Cu20 and Cu60. These results indicate that the oxidative stress caused by Cu toxicity induced the production of the two low-molecular-weight antioxidants and that the roots of Arabidopsis seedlings were more responsive to the oxidative stress.

**Figure 8:**
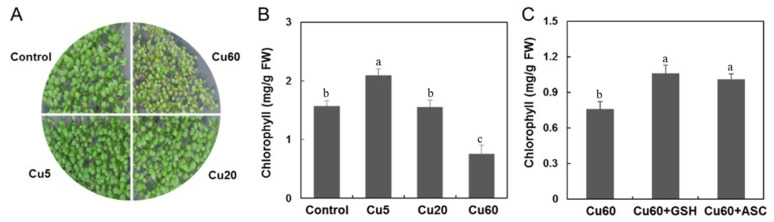
Effect of GSH and Asc on total chlorophyll of Arabidopsis seedlings exposed to Cu stress. Photos of 6-day-old Arabidopsis seedling treated with three different concentrations of CuSO_4_ (**A**). Total chlorophyll contents in leaves of different treatments (**B**). GSH and Asc treatments remedied the chlorosis of Cu-treated Arabidopsis seedlings (**C**). Data are means ± SD of three replicates with 30 seedlings. Different letters indicate the significant difference among treatments (*p* < 0.05, ANOVA-LSD post hoc test).

As a regulatory element of chlorophyll synthesis [51], excess Cu can lead to loss of chloroplast integrity and inhibit photosynthetic electron transport [35,52]. In this work, a significant decrease in chlorophyll content was observed on day 6, which was evident in the more yellowish leaves of Arabidopsis seedlings treated with Cu60 (Figure 8A,B). In contrast, the leaves treated with Cu5 and Cu20 had higher and similar total chlorophyll content, respectively, compared to the leaves of the control plants (Figure 8A,B), a result that differs from a previous report [35]. To further clarify the function of Asc and GSH in plant tolerance to excess Cu, we administered 0.3 mM Asc or 1 mM GSH in the presence of Cu60, both of which significantly increased the total chlorophyll content in treated leaves compared with plants treated with Cu60 alone (Figure 8C). It was suggested that exogenous Asc can replenish its endogenous pool and enhance photosynthetic electron transport [7]. Moreover, photosynthesis also depends on thiol-regulated enzymes, and GSH is involved in the thioredoxin regulation of many enzymes in photosynthetic metabolism [50]. The effects of exogenous GSH on heavy metal tolerance have been shown to depend on the plant species and the type of metal [53]. Exogenous GSH did not attenuate the toxicity of cadmium (Cd), copper (Cu), or zinc (Zn), whereas it significantly increased Hg tolerance during seed germination and seedling growth of *Arabidopsis thaliana* [54]. Treatment with GSH and Asc could also prevent chlorosis and the accumulation of ROS by increasing the activity of the heme protein ascorbate peroxidase, as reported in Arabidopsis leaves [55].

## 4. Conclusions

**Figure 9:**
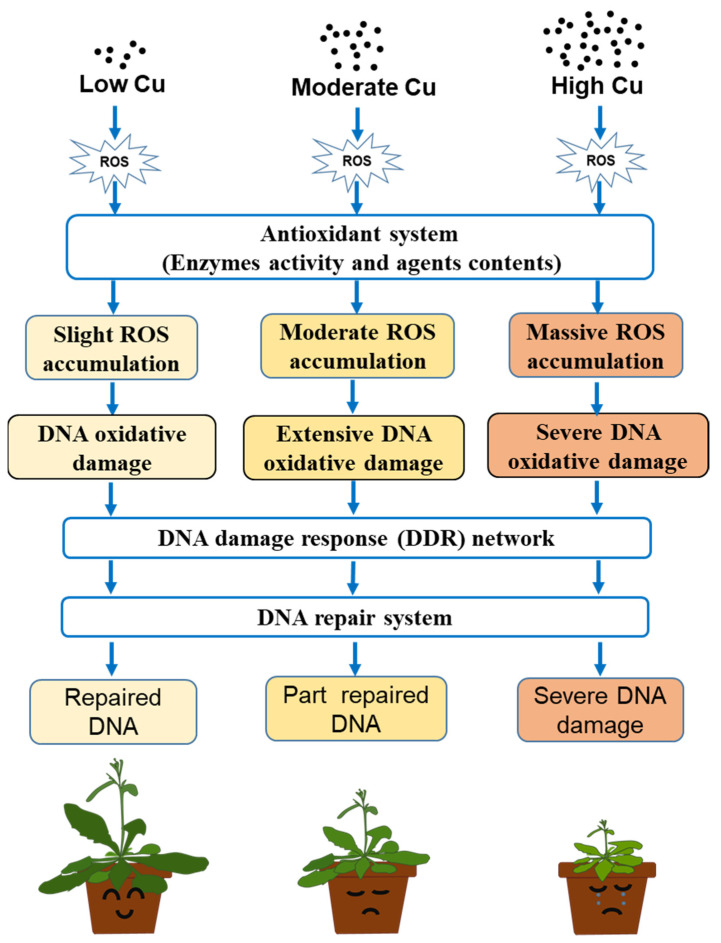
Graphical model of regulatory networks associated with DNA damage response and ROS homeostasis in dose-dependent responses of Arabidopsis seedlings to copper.

This work demonstrated the close relationship between the dose-dependent responses of Arabidopsis seedlings to Cu and altered endogenous ROS status. The development of oxidative stress, accumulation of oxidizing molecules, modified regulation of DDR, altered activities of antioxidant enzymes, and varying levels of low-molecular-weight antioxidants have been shown to contribute to the dose-dependent toxicity of Cu. In Figure 9, we present a graphical model that attempts to explain the various regulatory effects of increasing Cu concentrations, with signals generated by DNA damage and oxidative species providing the basis for the gene regulatory network to adjust counteracting ROS production and scavenging systems.

## Figures and Tables

**Table 1:** Reproductive growth characteristics of Arabidopsis plants grown in soil treated with three different concentrations of CuSO_4_. Data are means ± SD of three replicates. Different letters indicate a significant difference between treatments (*p* < 0.05, by ANOVA-LSD post hoc test).

CuSO_4_ (µM)	Control	Cu5	Cu20	Cu60
Stem height (cm)	39.0 ± 1.2 ^a^	39.1 ± 1.8 ^a^	39.1 ± 1.9 ^a^	38.6 ± 2.1 ^a^
Bolting time (d)	31.0 ± 2.0 ^b^	28.0 ± 3.0 ^c^	33.0 ± 3.0 ^b^	37.0 ± 2.0 ^a^
Silique lengths (cm)	1.2 ± 0.3 ^a^	1.3 ± 0.2 ^a^	1.1 ± 0.3 ^ab^	1.0 ± 0.1 ^b^
Thousand seed weight (mg DW)	18.9 ± 0.9 ^b^	21.9 ± 0.5 ^a^	19.9 ± 1.1 ^ab^	18.6 ± 0.6 ^b^

## Supplementary Materials

The following supporting information can be downloaded at: https://www.mdpi.com/article/10.3390/genes14010011/s1. Figure S1: Statistical significance results of NBT staining intensity in roots.; Figure S2: Statistical significance results of DAB staining intensity in roots. Figure S3: Statistical significance results of TB staining intensity in roots. Figure S4: Statistical significance results of O_2_^−^ content in roots. Figure S5: Statistical significance results of H_2_O_2_ contents in roots. Figure S6: Statistical significance results of MDA contents in roots. Figure S7: Statistical significance results of NBT staining intensity in shoots. Figure S8: Statistical significance results of DAB staining intensity in shoots. Figure S9: Statistical significance results of MDA contents in shoots. Figure S10: Statistical significance results of H_2_O_2_ contents in shoots. Figure S11: Statistical significance results of O_2_^−^ content in shoots. Figure S12: Statistical significance results of leaf electrolyte leakage. Table S1: Primers were used for qRT-PCR analysis.
