# Supplementary material for: ROS Homeostasis Involved in Dose-Dependent Responses of Arabidopsis Seedlings to Copper Toxicity
Source: Genes (Basel). 2022 Dec 21;14(1):11. doi: 10.3390/genes14010011 (PMC9858908; doi:10.3390/genes14010011)
Supplement: Supplementary file 1 [file genes-14-00011-s001.zip › genes-2088159-supplementary.pdf]

Supplementary Data

Table S1. Primers were used for qRT-PCR analysis.

Name	Gene Description	Locus	Forward primiers (5'-3')	Reverse primiers (5'-3')
BRCA1	Breast cancer susceptibility 1	AT4G21070	TTGCTCAGGGCTCACA	GGTCCTTTTGCAGGCT
RAD51	RAS associated with diabetes protein 51	AT5G20850	ATGAAGAAACCCAGCAC	TGAACCCCAGAGGAAC
MRE11	Meiotic recombination 11	AT5G54260	ACACTTCGAGTACTTGTTGC	ACTACTTGAAACTGCACTGG
WEE1	WEE1 kinase homolog	AT1G02970	GTGCTGGACATTTCAGTCGG	AGCTTGCACTTCCATCATAG
PARP1	Poly(ADP-ribose) polymerase 1	AT2G31320	TTGACGCCAGTAGGAA	AATACCAGCCCAGTTAG
MLH1	Mut1 DNA mismatch repair protein	AT4G09140	AGTAAGGTCTTCTGCAAGGCA	TGCCATTCCAACATATGTGC
MSH2	MutS homolog 2	AT3G18524	TCTGACTAGGCGAGTTCTT	CACCTCTCCAGGGAATCA
MSH6	MutS homolog 6	AT4G02070	TAGTTAGAAAGGGCTATCGGG	AACAACTGCACATACTTCGC
FSD1	Fe superoxide dismutase 1	AT4G25100	CTCAAGCCACCTCCATTCG	GCGTTGTTGAAAGCAGGGA
FSD2	Fe superoxide dismutase 2	AT5G51100	TGGATTATCACTGGGGCAAAC	GATAGACTCCCAGAAGAACTCG
FSD3	Fe superoxide dismutase 3	AT5G23310	GTGAACCCAACATCCCAATCG	CGTCACTAACATTACTGTCACC
CSD1	Copper/zinc superoxide dismutase 1	AT1G08830	ACCAAAGAGAGACGAAGCA	CTTCCTGGGTGAAAAAGATAG
CSD2	Copper/zinc superoxide dismutase 2	AT2G28190	AGGAGTTGTTACTTTGACCC	ACCACAAAGGCTCTTCCAAC
MSD1	Manganese superoxide dismutase 1	AT3G10920	GTTTGGGAGCACGCCTACTAC	CATCTCCTTATGTCATCGTGTA
CAT1	Catalase 1	AT1G20630	CGCCGATTTGCGAGATACA	ACCCTCTCAGGAATCCGCTC
CAT2	Catalase 2	AT4G35090	ATCCAACTCCGCCTGCTGTCT	ATGCGTGGGTCGGATAGGG
CAT3	Catalase 3	AT1G20620	GTGACACTCAGAGACATCGCC	AAACCTGTCTTGCCTGTCTGG
APX1	Ascorbate peroxidase	AT1G07890	ATACGCTGCTGATGAAGATG	AGACACACACACACACACAG
GPX1	Glutathione peroxidase	AT2G25080	ACCGTTCACGATTTCACC	ACCACCCAAGAATCCTCC
VTC1	Vitamin C defective 1	AT2G39770	ATCACGGCTATACACGG	AGAAGTTAAAGCTGGAACCGC
GSH1	Glutathione synthetase 1	AT4G23100	ATCTACGCTTTGTCCCCATTC	ATATTCCCAGAGGTTCGGTG
GSH2	Glutathione synthetase 2	AT5G27380	ACCAACTGCATTCCCAGAAG	GCCATCCAAGCTAACACGA
Actin	Actin 12	AT3G46520	TGGTGGTGAATGAGTAGCC	AGCAAACAGAGAAAAGATGA

Figure S1. Statistical significance results of NBT staining intensity in roots.

Multiple Comparisons

Dependent Variable: Content

LSD

(I) Cu	(J) Cu	Mean Difference (I-J)	Std. Error	Sig.	95% Confidence Interval Lower Bound	95% Confidence Interval Upper Bound
0	5	-45.32667*	3.76083	<.001	-52.9061	-37.7472
	20	-85.22917*	3.76083	<.001	-92.8086	-77.6497
	60	-131.37917*	3.76083	<.001	-138.9586	-123.7997
5	0	45.32667*	3.76083	<.001	37.7472	52.9061
	20	-39.90250*	3.76083	<.001	-47.4820	-32.3230
	60	-86.05250*	3.76083	<.001	-93.6320	-78.4730
20	0	85.22917*	3.76083	<.001	77.6497	92.8086
	5	39.90250*	3.76083	<.001	32.3230	47.4820
	60	-46.15000*	3.76083	<.001	-53.7295	-38.5705
60	0	131.37917*	3.76083	<.001	123.7997	138.9586
	5	86.05250*	3.76083	<.001	78.4730	93.6320
	20	46.15000*	3.76083	<.001	38.5705	53.7295

*. The mean difference is significant at the 0.05 level.

Figure S2. Statistical significance results of DAB staining intensity in roots.

Multiple Comparisons

Dependent Variable: Content

LSD

(I) Cu	(J) Cu	Mean Difference (I-J)	Std. Error	Sig.	95% Confidence Interval Lower Bound	95% Confidence Interval Upper Bound
0	5	-16.36500*	3.41067	<.001	-23.2388	-9.4912
	20	-61.33333*	3.41067	<.001	-68.2071	-54.4596
	60	-152.79000*	3.41067	<.001	-159.6638	-145.9162
5	0	16.36500*	3.41067	<.001	9.4912	23.2388
	20	-44.96833*	3.41067	<.001	-51.8421	-38.0946
	60	-136.42500*	3.41067	<.001	-143.2988	-129.5512
20	0	61.33333*	3.41067	<.001	54.4596	68.2071
	5	44.96833*	3.41067	<.001	38.0946	51.8421
	60	-91.45667*	3.41067	<.001	-98.3304	-84.5829
60	0	152.79000*	3.41067	<.001	145.9162	159.6638
	5	136.42500*	3.41067	<.001	129.5512	143.2988
	20	91.45667*	3.41067	<.001	84.5829	98.3304

*. The mean difference is significant at the 0.05 level.

Figure S3. Statistical significance results of TB staining intensity in roots.

Multiple Comparisons

Dependent Variable: Content

LSD

(I) Cu	(J) Cu	Mean Difference (I-J)	Std. Error	Sig.	95% Confidence Interval	
					Lower Bound	Upper Bound
0	5	-15.82917*	2.21695	<.001	-20.2971	-11.3612
	20	-43.81833*	2.21695	<.001	-48.2863	-39.3504
	60	-62.31333*	2.21695	<.001	-66.7813	-57.8454
5	0	15.82917*	2.21695	<.001	11.3612	20.2971
	20	-27.98917*	2.21695	<.001	-32.4571	-23.5212
	60	-46.48417*	2.21695	<.001	-50.9521	-42.0162
20	0	43.81833*	2.21695	<.001	39.3504	48.2863
	5	27.98917*	2.21695	<.001	23.5212	32.4571
	60	-18.49500*	2.21695	<.001	-22.9630	-14.0270
60	0	62.31333*	2.21695	<.001	57.8454	66.7813
	5	46.48417*	2.21695	<.001	42.0162	50.9521
	20	18.49500*	2.21695	<.001	14.0270	22.9630

*. The mean difference is significant at the 0.05 level.

Figure S4. Statistical significance results of O_2^- content in roots.

Multiple Comparisons

Dependent Variable: Content

LSD

(I) Cu	(J) Cu	Mean Difference (I-J)	Std. Error	Sig.	95% Confidence Interval	
					Lower Bound	Upper Bound
0	5	-.67500*	.12781	<.001	-.9342	-.4158
	20	-1.95900*	.12781	<.001	-2.2182	-1.6998
	60	-2.84700*	.12781	<.001	-3.1062	-2.5878
5	0	.67500*	.12781	<.001	.4158	.9342
	20	-1.28400*	.12781	<.001	-1.5432	-1.0248
	60	-2.17200*	.12781	<.001	-2.4312	-1.9128
20	0	1.95900*	.12781	<.001	1.6998	2.2182
	5	1.28400*	.12781	<.001	1.0248	1.5432
	60	-.88800*	.12781	<.001	-1.1472	-.6288
60	0	2.84700*	.12781	<.001	2.5878	3.1062
	5	2.17200*	.12781	<.001	1.9128	2.4312
	20	.88800*	.12781	<.001	.6288	1.1472

*. The mean difference is significant at the 0.05 level.

Figure S5. Statistical significance results of H_2O_2 contents in roots.

Multiple Comparisons

Dependent Variable: Content

LSD

(I) Cu	(J) Cu	Mean Difference (I-J)	Std. Error	Sig.	95% Confidence Interval Lower Bound	95% Confidence Interval Upper Bound
0	5	5.12200	3.04665	.101	-1.0569	11.3009
	20	-40.09800*	3.04665	<.001	-46.2769	-33.9191
	60	-105.39600*	3.04665	<.001	-111.5749	-99.2171
5	0	-5.12200	3.04665	.101	-11.3009	1.0569
	20	-45.22000*	3.04665	<.001	-51.3989	-39.0411
	60	-110.51800*	3.04665	<.001	-116.6969	-104.3391
20	0	40.09800*	3.04665	<.001	33.9191	46.2769
	5	45.22000*	3.04665	<.001	39.0411	51.3989
	60	-65.29800*	3.04665	<.001	-71.4769	-59.1191
60	0	105.39600*	3.04665	<.001	99.2171	111.5749
	5	110.51800*	3.04665	<.001	104.3391	116.6969
	20	65.29800*	3.04665	<.001	59.1191	71.4769

*. The mean difference is significant at the 0.05 level.

Figure S6. Statistical significance results of MDA contents in roots.

Multiple Comparisons

Dependent Variable: Content

LSD

(I) Cu	(J) Cu	Mean Difference (I-J)	Std. Error	Sig.	95% Confidence Interval Lower Bound	95% Confidence Interval Upper Bound
0	5	-.14400	.15038	.345	-.4490	.1610
	20	-.97900*	.15038	<.001	-1.2840	-.6740
	60	-1.71600*	.15038	<.001	-2.0210	-1.4110
5	0	.14400	.15038	.345	-.1610	.4490
	20	-.83500*	.15038	<.001	-1.1400	-.5300
	60	-1.57200*	.15038	<.001	-1.8770	-1.2670
20	0	.97900*	.15038	<.001	.6740	1.2840
	5	.83500*	.15038	<.001	.5300	1.1400
	60	-.73700*	.15038	<.001	-1.0420	-.4320
60	0	1.71600*	.15038	<.001	1.4110	2.0210
	5	1.57200*	.15038	<.001	1.2670	1.8770
	20	.73700*	.15038	<.001	.4320	1.0420

*. The mean difference is significant at the 0.05 level.

Figure S7. Statistical significance results of NBT staining intensity in shoots.

Multiple Comparisons

Dependent Variable: Content

LSD

(I) Cu	(J) Cu	Mean Difference (I-J)	Std. Error	Sig.	95% Confidence Interval Lower Bound	95% Confidence Interval Upper Bound
0	5	-42.53250*	3.96675	<.001	-50.5270	-34.5380
	20	-68.47750*	3.96675	<.001	-76.4720	-60.4830
	60	-134.74583*	3.96675	<.001	-142.7403	-126.7514
5	0	42.53250*	3.96675	<.001	34.5380	50.5270
	20	-25.94500*	3.96675	<.001	-33.9395	-17.9505
	60	-92.21333*	3.96675	<.001	-100.2078	-84.2189
20	0	68.47750*	3.96675	<.001	60.4830	76.4720
	5	25.94500*	3.96675	<.001	17.9505	33.9395
	60	-66.26833*	3.96675	<.001	-74.2628	-58.2739
60	0	134.74583*	3.96675	<.001	126.7514	142.7403
	5	92.21333*	3.96675	<.001	84.2189	100.2078
	20	66.26833*	3.96675	<.001	58.2739	74.2628

*. The mean difference is significant at the 0.05 level.

Figure S8. Statistical significance results of DAB staining intensity in shoots.

Multiple Comparisons

Dependent Variable: Content

LSD

(I) Cu	(J) Cu	Mean Difference (I-J)	Std. Error	Sig.	95% Confidence Interval Lower Bound	95% Confidence Interval Upper Bound
0	5	-16.75917*	1.80899	<.001	-20.4049	-13.1134
	20	-28.87833*	1.80899	<.001	-32.5241	-25.2326
	60	-59.17583*	1.80899	<.001	-62.8216	-55.5301
5	0	16.75917*	1.80899	<.001	13.1134	20.4049
	20	-12.11917*	1.80899	<.001	-15.7649	-8.4734
	60	-42.41667*	1.80899	<.001	-46.0624	-38.7709
20	0	28.87833*	1.80899	<.001	25.2326	32.5241
	5	12.11917*	1.80899	<.001	8.4734	15.7649
	60	-30.29750*	1.80899	<.001	-33.9433	-26.6517
60	0	59.17583*	1.80899	<.001	55.5301	62.8216
	5	42.41667*	1.80899	<.001	38.7709	46.0624
	20	30.29750*	1.80899	<.001	26.6517	33.9433

*. The mean difference is significant at the 0.05 level.

Figure S9. Statistical significance results of MDA contents in shoots.

Multiple Comparisons

Dependent Variable: Content

LSD

(I) Cu	(J) Cu	Mean Difference (I-J)	Std. Error	Sig.	95% Confidence Interval	
					Lower Bound	Upper Bound
0	5	-.71200*	.20631	.001	-1.1304	-.2936
	20	-2.65700*	.20631	<.001	-3.0754	-2.2386
	60	-5.15300*	.20631	<.001	-5.5714	-4.7346
5	0	.71200*	.20631	.001	.2936	1.1304
	20	-1.94500*	.20631	<.001	-2.3634	-1.5266
	60	-4.44100*	.20631	<.001	-4.8594	-4.0226
20	0	2.65700*	.20631	<.001	2.2386	3.0754
	5	1.94500*	.20631	<.001	1.5266	2.3634
	60	-2.49600*	.20631	<.001	-2.9144	-2.0776
60	0	5.15300*	.20631	<.001	4.7346	5.5714
	5	4.44100*	.20631	<.001	4.0226	4.8594
	20	2.49600*	.20631	<.001	2.0776	2.9144

*. The mean difference is significant at the 0.05 level.

Figure S10. Statistical significance results of H_2O_2 contents in shoots.

Multiple Comparisons

Dependent Variable: Content

LSD

(I) Cu	(J) Cu	Mean Difference (I-J)	Std. Error	Sig.	95% Confidence Interval	
					Lower Bound	Upper Bound
0	5	-1.55308*	.68689	.028	-2.9342	-.1720
	20	-3.22538*	.68689	<.001	-4.6065	-1.8443
	60	-11.40538*	.68689	<.001	-12.7865	-10.0243
5	0	1.55308*	.68689	.028	.1720	2.9342
	20	-1.67231*	.68689	.019	-3.0534	-.2912
	60	-9.85231*	.68689	<.001	-11.2334	-8.4712
20	0	3.22538*	.68689	<.001	1.8443	4.6065
	5	1.67231*	.68689	.019	.2912	3.0534
	60	-8.18000*	.68689	<.001	-9.5611	-6.7989
60	0	11.40538*	.68689	<.001	10.0243	12.7865
	5	9.85231*	.68689	<.001	8.4712	11.2334
	20	8.18000*	.68689	<.001	6.7989	9.5611

*. The mean difference is significant at the 0.05 level.

Figure S11. Statistical significance results of O_2^- content in shoots.

Multiple Comparisons

Dependent Variable: Content

LSD

(I) Cu	(J) Cu	Mean Difference (I-J)	Std. Error	Sig.	95% Confidence Interval Lower Bound	95% Confidence Interval Upper Bound
0	5	-.77800*	.12767	<.001	-1.0369	-.5191
	20	-3.28900*	.12767	<.001	-3.5479	-3.0301
	60	-5.21600*	.12767	<.001	-5.4749	-4.9571
5	0	.77800*	.12767	<.001	.5191	1.0369
	20	-2.51100*	.12767	<.001	-2.7699	-2.2521
	60	-4.43800*	.12767	<.001	-4.6969	-4.1791
20	0	3.28900*	.12767	<.001	3.0301	3.5479
	5	2.51100*	.12767	<.001	2.2521	2.7699
	60	-1.92700*	.12767	<.001	-2.1859	-1.6681
60	0	5.21600*	.12767	<.001	4.9571	5.4749
	5	4.43800*	.12767	<.001	4.1791	4.6969
	20	1.92700*	.12767	<.001	1.6681	2.1859

*. The mean difference is significant at the 0.05 level.

Figure S12. Statistical significance results of leaf electrolyte leakage.

Multiple Comparisons

Dependent Variable: Content

LSD

(I) Cu	(J) Cu	Mean Difference (I-J)	Std. Error	Sig.	95% Confidence Interval Lower Bound	95% Confidence Interval Upper Bound
0	5	-1.71300	1.20753	.165	-4.1620	.7360
	20	-10.74800*	1.20753	<.001	-13.1970	-8.2990
	60	-20.94900*	1.20753	<.001	-23.3980	-18.5000
5	0	1.71300	1.20753	.165	-.7360	4.1620
	20	-9.03500*	1.20753	<.001	-11.4840	-6.5860
	60	-19.23600*	1.20753	<.001	-21.6850	-16.7870
20	0	10.74800*	1.20753	<.001	8.2990	13.1970
	5	9.03500*	1.20753	<.001	6.5860	11.4840
	60	-10.20100*	1.20753	<.001	-12.6500	-7.7520
60	0	20.94900*	1.20753	<.001	18.5000	23.3980
	5	19.23600*	1.20753	<.001	16.7870	21.6850
	20	10.20100*	1.20753	<.001	7.7520	12.6500

*. The mean difference is significant at the 0.05 level.
